# Perseverative Cognition in the Positive Valence Systems: An Experimental and Ecological Investigation

**DOI:** 10.3390/brainsci11050585

**Published:** 2021-04-30

**Authors:** Martino Schettino, Valerio Ghezzi, Yuen-Siang Ang, Jessica M. Duda, Sabrina Fagioli, Douglas S. Mennin, Diego A. Pizzagalli, Cristina Ottaviani

**Affiliations:** 1 Department of Psychology, Sapienza University of Rome, 00185 Rome, Italy; valerio.ghezzi@uniroma1.it; 2 Department of Social and Cognitive Computing, Institute of High Performance Computing, Agency for Science, Technology and Research, Singapore 138632, Singapore; angys@ihpc.a-star.edu.sg; 3 Center for Depression, Anxiety and Stress Research, McLean Hospital, Belmont, MA 02478, USA; JDUDA1@mclean.harvard.edu (J.M.D.); dap@mclean.harvard.edu (D.A.P.); 4 Department of Education, University of Roma Tre, 00185 Rome, Italy; sabrina.fagioli@uniroma3.it; 5 Teachers College, Columbia University, New York, NY 10027, USA; dm3297@tc.columbia.edu; 6 Department of Psychiatry, Harvard Medical School, Belmont, MA 02115, USA; 7 Neuroimaging Laboratory, IRCCS Santa Lucia Foundation, 00179 Rome, Italy

**Keywords:** perseverative cognition, positive valence systems, RDoC, probabilistic reward task, ecological momentary assessment, reward prediction error, transdiagnostic psychiatry

## Abstract

Perseverative cognition (PC) is a transdiagnostic risk factor that characterizes both hypo-motivational (e.g., depression) and hyper-motivational (e.g., addiction) disorders; however, it has been almost exclusively studied within the context of the negative valence systems. The present study aimed to fill this gap by combining laboratory-based, computational and ecological assessments. Healthy individuals performed the Probabilistic Reward Task (PRT) before and after the induction of PC or a waiting period. Computational modeling was applied to dissociate the effects of PC on reward sensitivity and learning rate. Afterwards, participants underwent a one-week ecological momentary assessment of daily PC occurrence, as well as anticipatory and consummatory reward-related behavior. Induction of PC led to increased response bias on the PRT compared to waiting, likely due to an increase in learning rate but not in reward sensitivity, as suggested by computational modeling. In daily-life, PC increased the discrepancy between expected and obtained rewards (i.e., prediction error). Current converging experimental and ecological evidence suggests that PC is associated with abnormalities in the functionality of positive valence systems. Given the role of PC in the prediction, maintenance, and recurrence of psychopathology, it would be clinically valuable to extend research on this topic beyond the negative valence systems.

## 1. Introduction

A transdiagnostic approach in psychiatry promises to overcome the limits of categorial diagnostic classification systems of mental illness and to improve clinical care and treatment [1]. Perseverative cognition (PC [2]) is a form of cognition characterized by repetitive, intrusive and generally negative (e.g., worrisome and ruminative) thoughts, and it is now recognized as a transdiagnostic factor for the onset, maintenance and recurrence of psychiatric disorders [3]. Different forms of PC are common across distinct neuropsychiatric disorders, including depression and anxiety disorders [4], post-traumatic stress disorder [5], eating disorders [6], and substance abuse disorders [7].

Despite significant efforts to understand PC both at a psychological and biological level in individuals with and without a psychiatric condition, the mechanisms by which PC contributes to the onset and maintenance of psychopathology remain unclear. A partial explanation stems from the fact that the pathophysiology of PC has been investigated using a specific-disorder approach (e.g., PC in depression [8]), instead of approaching it as a dimensional and transdiagnostic construct.

The Research Domain Criteria (RDoC), a National Institute of Mental Health proposal linking genetic, neuroscience and behavioral science constructs relevant to psychopathology [9], is a useful tool to create a cohesive mechanistic understanding of PC. In terms of RDoC, worry and rumination are referred to in the functionality of the negative valence systems domain. Endorsing, recent physiological, neuroimaging, behavioral and clinical data have supported an overlap between PC and several measures of the loss construct [10,11,12,13]. However, as previously noted, PC is also present in disorders such as substance use or in the manic phase of bipolar disorder [14,15], pointing to a potential gap in understanding the effect of PC on the positive valence systems domain. Indeed, endorsing a recent proposal for innovation within RDoC to include “cross-construct patterns of dimensional measurements both across and within different RDoC domains to specify agnostic and possibly transdiagnostic subtypes of psychiatric illnesses” (page 308, [16]), it would be important to assess PC-inducing effects on the positive valence systems. In general, the positive valence systems domain involves the regulation of behaviors that result in reward achievement, such as reward responsiveness and learning.

A well-validated task to measure reward responsiveness and reward learning included in the RDoC matrix is the Probabilistic Reward Task [17], which provides an objective assessment of the ability to modulate behavior as a function of prior reinforcement. The PRT has been used across laboratories to provide evidence for acute stress-related blunting of reward responsiveness and reward learning. For example, in early studies, threat of shock—but not negative performance feedback—was found to reduce the ability to modulate behavior as a function of past reward in non-pathological individuals [18,19]. Given that PC has been conceptualized as a prolonged cognitive and physiological stress response (see [20] for a meta-analysis), one would expect to observe impaired (i.e., blunted) reward responsiveness and reward learning during episodes of PC.

Existing studies on this issue are sparse and limited by the focus on PC as a trait, assessed by retrospective self-report questionnaires. Keeping in mind these limitations, it is noteworthy that such studies all present a somewhat opposite picture. For example, in a study examining the dispositional tendency to ruminate, positive associations between scores on the Rumination Response Scale [21] and the following were reported: (i) activation of the ventral striatum in response to rewards; and (ii) a ruminative-dependent resting state increased functional connectivity in the cortico-striatal circuits [22]. In an earlier study, the induction of rumination—as opposed to distraction—similarly led to greater sensitivity to reward probabilities, assessed with the Probabilistic Selection Task [23], in a a group of individuals with depression and controls [24]. It should be noted, however, that, in the study by Erdman et al. [22], rumination was not experimentally manipulated, thus precluding any causal inference on the effects of PC on the neural responses to rewards. Similarly, in the study by Whitmer et al. [24], the induction exclusively focused on depressive rumination, rather than on the transdiagnostic construct of PC, and the between-subjects study design did not allow any inferences about the pre- to post-manipulation changes to be made.

The current study sought to investigate the effects of PC on positive valence systems functionality, overcoming the two aforementioned limitations of previous studies. Toward this aim, an experimental study was conducted to investigate the effects of a PC manipulation on behavioral markers of reward responsiveness and reward learning in a sample of psychiatrically healthy individuals. Given the limited generalizability of experimental settings when studying motivational processes and spontaneous phenomenon such as PC, our second goal was to investigate the ability to respond to and learn from rewards during episodes of daily-life PC.

To this end, using a mixed design, we first used a well-replicated induction of PC (for a review, see [25]), which was administered between two PRT assessments in a sample of healthy individuals. Participants were selected to be normally distributed on their levels of dispositional tendency to engage in PC, based on the Perseverative Thinking Questionnaire (PTQ [26]), which provides a measure of transdiagnostic and content-independent processes underpinning PC. To parse the contribution of learning rate (operationalized as participants’ ability to accumulate rewards over time and learn from the reward) and reward sensitivity (operationalized as consummatory pleasure or the immediate behavioral impact of rewards) on PRT performance, a computational model of trial-level performance was also implemented. Then, we adapted a recently developed ecological momentary assessment (EMA [27]), specifically hypothesized to capture a daily-life motivational system functionality [28], to study the association between PC and reward processing in everyday life in the same group of participants over a week.

Based on the previously reported effects of stress induction on reward-related behavior, we hypothesized that state PC would impair reward responsiveness, as manifested in both the experimental (i.e., impaired performance on the PRT) and the ecological (i.e., reduced momentary reward anticipation and pleasantness) paradigms.

## 2. Materials and Methods

### 2.1. Participants

Participants were recruited at undergraduate psychology degree courses, and through word of mouth. They were invited to participate in a study on “attentional processes, mood and response to rewards”, and were told that they could win up to 30 euros during the experiment. Participants were enrolled after providing a written informed consent to a protocol approved by the Institutional Review board of the Department of Psychology (Ref. N. 740/2020). Exclusionary criteria were history or presence of serious medical or psychiatric conditions and use of drugs/medications.

An a priori power analysis was performed with G Power 3 [29] based on the effect size found in previous studies for one of the variables of interest (ΔResponse Bias). With power of 1−β > 0.90 and a significance value of *p* < 0.05, the power analysis revealed that, in a mixed-design with the between-subject factor Group and the within-subject factor Time, an adequate number of participants would be *n* = 29 per group. In total, 55 healthy participants met eligibility criteria during recruitment. Among them, six participants were excluded from PRT analyses because they did not complete the task post- manipulation. Among the remaining 49, three participants did not pass the quality control check on PRT performance, which was performed blind to group assignment using predefined cut-off scores (see Supplementary Materials). Thus, for the experimental component of the study, data from 46 participants were available for the analysis (35 women, 11 men; 22.96 ± 5.33 years). With regards to the Ecological Momentary Assessment (EMA) component of the study, exclusion criteria were defined as follows: participants should have provided valid observations: (1) for more than a day; and (2) higher than the 20% of the total e-mailed “beeps”. Three participants were dropped from these analyses because they did not fulfill both inclusion criteria. Accordingly, the final sample for the EMA study comprised 52 participants (39 women and 13 men).

### 2.2. Procedure

The study was entirely implemented online, due to the SARS-CoV-2 pandemic. First, participants received the written informed consent, and, once signed, a series of dispositional questionnaires via the survey software Qualtrics (https://www.qualtrics.com, accessed on 29 April 2021). Participants were semi-randomly assigned to the experimental or control groups based on their scores on the PTQ: specifically, PTQ scores were balanced both within and across groups with the same number of participants belonging to each tertile in each group. After filling out the questionnaires, participants were invited to perform an online attention task and an appointment was scheduled. The Millisecond version of the PRT was adapted following the authors’ guidelines and administered via Inquisit Web (https://www.millisecond.com/products/inquisit6/weboverview.aspx, accessed on 29 April 2021), a tool to collect behavioral data remotely (see Supplementary Materials). An Inquisit script was programmed to start with the assessment of mood and baseline levels of PC, by the use of Visual-Analog Scales (VASs). Then, all participants underwent two sessions of the PRT (mouth and nose version), before and after a 2-min induction of PC (experimental group) or a 2-min wait (control group). Assessment of mood and PC were repeated after the experimental manipulation, and again at the end of the second PRT. This session was followed by a 7-day ecological momentary assessment. After one week, participants were fully debriefed and compensated for participation with the money that they won during the task.

### 2.3. Questionnaires

All participants completed a set of questions assessing sociodemographic and lifestyle or medical information (i.e., nicotine, alcohol and caffeine consumption and medication intake). To obtain a measure of dispositional tendency to engage in some forms of PC, participants were administered the PTQ [26], a 15-item questionnaire developed to capture the processes typical of repetitive thinking instead of the specific content of thoughts, namely repetitiveness (e.g., “The same thoughts keep going through my mind again and again”), intrusiveness (e.g., “Thoughts come to my mind without me wanting them to”), difficulties in disengaging (e.g., “I can’t stop dwelling on them”), unproductiveness (e.g., “I keep asking myself questions without finding an answer”), and mental capacity absorbing (e.g., “My thought prevent me from focusing on other things”).

Additional dispositional traits were assessed to control for potential between-groups baseline differences: (a) Behavioral inhibition and behavioral activation Scale (BIS/BAS [30]), Cronbach’s alpha in the present sample = 0.73; (b) Barratt Impulsiveness Scale (BIS-11 [31]), Cronbach’s alpha in the present sample = 0.83; (c) Snaith–Hamilton pleasure scale (SHAPS [32]), Cronbach’s alpha in the present sample = 0.84; (d) State-Trait Anxiety Inventory (STAI; [33]), Cronbach’s alpha in the present sample = 0.93; (e) Center for Epidemiologic Studies Depression Scale (CES-D [34]), Cronbach’s alpha in the present sample = 0.72; (f) Optimism scale [35]; and (g) Sensation Seeking Scale V [36], Cronbach’s alpha in the present sample = 0.91.

### 2.4. Perseverative Cognition Induction

A well-replicated verbal induction procedure designed to induce PC was used (see [25] for a systematic review). Specifically, participants were asked to tell the experimenter (for 2 min) about a negative personal episode that happened within the past year that made them feel sad, anxious, or stressed and—when thinking about it—still made them sad, anxious, or stressed or something that may happen in the future that worried them (see Supplementary Materials). Participants in the control group were asked to wait until the next instructions (2 min).

### 2.5. Visual-Analog Scales

Visual-Analog Scales (VAS) were administered at the beginning of the protocol (VAS 1), after performance on the first PRT (VAS 2), after the induction (VAS 3), and after performance on the second PRT (VAS 4) to assess manipulation effects on self-reported momentary mood and levels of state PC. Momentary mood was assessed by the questions “In this moment how much do you feel: happy/anxious/satisfied/sad/energetic”. To assess levels of state PC before and after the induction or control condition, participants were asked “In this moment how much do you feel” (1) “distracted by thoughts/past memories/future worries/personal problems”; and (2) “stuck on these thoughts/past memories/future worries/personal problems without being able to think about anything else?”

### 2.6. Probabilistic Reward Task

The PRT is a task designed to yield an objective measure of participants’ ability to modify their behavior as a function of a reward and provides objective measure of reward responsiveness and reward learning [17]. On each trial, participants were asked to decide whether a long or short stimulus (mouth or nose) was presented (for 100 ms) on a cartoon face by pressing one of two fixed buttons on the keyboard (“E” and “I”). Importantly, and in line with previous studies in healthy participants (e.g., [18]), the difference between the two stimuli was very subtle (10–11 mm for the mouth version and 5–5.31 mm for the nose version). Of note, and unbeknownst to the participants, one stimulus (called “rich stimulus”) was rewarded more frequently compared to the other (called “lean stimulus”), according to a 3-to-1 asymmetrical reinforcement ratio in favor of the rich stimulus (see Supplementary Materials for more detailed information). Thus, reward learning is implicit and based on the reinforcement history rather than on the perceptual differences between the two stimuli. Each participant completed the task twice: before and after a PC induction or waiting. To reduce carry-over effects between conditions (pre-post), the mouth and nose versions of the PRT were utilized, before and after the experimental manipulation, respectively (i.e., not counterbalanced across subjects). The target of the asymmetrical reinforcement ratio and the button on keyboard to press for long or short stimulus were counterbalanced within and between participants for a total of four different combinations.

### 2.7. Data Reduction

According to signal detection theory, the main variables of interest were: (1) response bias, an empirically derived measure of systematic preference to choose the most frequently rewarded stimulus; and (2) discriminability, which provides a control measure of participants’ ability to discriminate between the two stimuli and reflects task difficulty. According to an established procedure [17,18,37], response bias (log b) and discriminability (log d) were calculated, respectively, as:

(1) $$logb=\frac{1}{2}log\left[\frac{\left(RICHcorrect+0.5\right)\times \left(LEANincorrect+0.5\right)}{\left(RICHincorrect+0.5\right)\times \left(LEANcorrect+0.5\right)}\right],$$

(2) $$logd=\frac{1}{2}log\left[\frac{\left(RICHcorrect+0.5\right)\times \left(LEANcorrect+0.5\right)}{\left(RICHincorrect+0.5\right)\times \left(LEANincorrect+0.5\right)}\right],$$

where *RICHcorrect* and *RICHincorrect* are the number of the correct and incorrect responses to the rich stimulus, respectively, and *LEANcorrect* and *LEANincorrect* are those to the lean stimulus. As evidenced in Equation (1), a higher response bias results from a larger numerator (i.e., larger number of rich correct and lean incorrect) or from a smaller denominator (i.e., smaller number of rich correct and lean correct). As done in previous studies using this task [38,39], 0.5 was added to each variable to make the calculation of the response bias and discriminability possible in cases in which one of the raw cells was equal to 0.

To further evaluate behavioral responses, the percentage of correct responses (i.e., accuracy) for each stimulus (rich or lean) type was calculated.

### 2.8. Computation Modeling of PRT

A series of reinforcement learning models were fitted to participants’ trial-by-trial data according to previously established procedures (see [40] for details). The ‘Stimulus-Action’ model treated both stimuli as being completely distinct and associated rewards with stimulus-action pairs. In the ‘Action’ model, subjects were assumed to neglect the stimuli and only learned action values when forming expectations. Another model, ‘Belief’, proposed that rewards were associated with a mixture of two stimulus-action associations weighted by an uncertainty factor. Lastly, a ‘Punishment’ model evaluated whether zero rewards were regarded as aversive losses. Expectation-maximization [41] was utilized to derive group priors in these models and individual Laplace approximation of posterior distributions was used to estimate parameters for every subject (see Supplementary Methods). Model comparisons were conducted via integrated group-level Bayesian Information Criterion factors. The most parsimonious account of the data was provided by the ‘Action’ model, with a group-level log Bayes factor compared to second-best ‘Belief’ model of 68 (which represents very strong evidence). This approach allowed two main parameters to be derived: reward sensitivity, which assessed the immediate behavioral impact of rewards (mean = 0.887, SD = 0.349), and learning rate (mean = −5.17, SD = 1.52), which measured ability to learn from rewards accumulated over time. Note that parameters were computed in the transformed space in order to prevent issues with non-Gaussianity. Hence, learning rate is no longer constrained to the range of 0–1 and reward sensitivity no longer constrained to range of 0 to +inf. Instead, both parameters are unconstrained from –inf to +inf, but larger values still indicate greater learning rate and reward sensitivity. Two other parameters that are not the focus of this study were also obtained: instruction sensitivity (mean = 0.167, SD = 0.778) and initial bias (mean = −0.018, SD = 0.209).

### 2.9. EMA

To measure reward responsiveness during daily life events of PC, an ecological momentary assessment with a time-contingent design [42] was implemented in Qualtrics. An array of emails was scheduled to arrive at unpredictable moments about every 120 min, between 10:00 a.m. and 10:30 p.m. for a period of 7 days (i.e., 7 e-mails per day). Each e-mail had a link to an electronic diary which took about 2–3 min to be filled in. The diary was available for a maximum of 20 min from the initial notification, but participants were asked to fill out the questions as soon as possible to reduce recall bias.

#### EMA Measures

The items were created to measure the processes underlying PC (intensity, intrusiveness, repetitiveness and stuck), rather than the specific content of the perseverative thoughts [43]. Participants had to rate on a 7-point Likert scale (from Not at all to Very much): (1) “Right now, how much were you distracted by your thoughts (i.e., past memories, future worries, personal problems)?”; (2) “How much were these thoughts going through your mind again and again?”; (3) “How much were these thoughts coming to your mind without you wanting them to?”; and (4) “How much were you stuck on certain issues and could not move on?”. Due to the high correlation between Items 2 and 3, Item 3 was dropped and the final PC composited was obtained by summing the three scores (Items 1, 2, and 4) per participant and time point of assessment.

According to one of the few previous studies on reward-related behavior in daily life [28], probabilistic reward-related behavior was measured on a 7-point Likert scale (from Not at all to Very much) by questions assessing: (1) Reward anticipation, “Think about what you consider to be the most rewarding situation in the next hour...How much are you looking forward to it?”; (2) Effort, “How much effort are you willing to exert to make it happen?”; and (3) Activity pleasantness, “Think about what you were doing right before receiving the email...How much did you feel actively engaged in such activity?” “How much were you enjoying it?”. As above for PC, composite for Activity Pleasantness was created by summing the scores of their relative items per participants and time point of assessment.

### 2.10. Data Analysis

#### 2.10.1. Experimental Session

Preliminary evaluations were conducted to ensure no violation of the assumptions of normality, linearity, homogeneity of variances, and sphericity. Series of independent sample *t* and $\chi^{2}$ tests were performed to exclude the presence of pre-existing socio-demographic (age, sex, medication use, and smoking), dispositional (scores on the questionnaires), and affective state (scores on the VAS at baseline) differences between the two groups.

To evaluate the efficacy of the induction procedure in eliciting PC, change scores were computed using scores on the VAS post-manipulation (VAS 3) minus scores pre-manipulation (VAS 2) and performing a series of independent sample *t*-tests. Then, to test whether the effects of the induction continued throughout performance on the second PRT, change scores were computed using scores on the VAS at the end of the protocol (VAS 4) minus post-manipulation scores (VAS 3) and a series of independent sample *t*-tests were performed.

Two General Linear Models (GLMs), with Block (Block 1, Block 2, Block 3) and Time (pre, post) as within-subject variables and Group (experimental, control) as a between-subject variable, were computed separately on response bias and discriminability. For accuracy, the GLM also included the within-subjects Stimulus Type (lean, rich). Then, to assess the magnitude of change in reinforcement learning before and after the experimental manipulation, a GLM with Time (pre, post) and Group (experimental, control) was run on ΔResponse Bias, computed as Response Bias during Block 3 minus Response Bias during Block 1, as in previous studies (see, e.g., [38]). Lastly, two separate GLMs with Time and Group were performed on computational parameters of reward sensitivity and learning rate.

#### 2.10.2. Ecological Momentary Assessment (EMA)

First, we evaluated empirically the missing data mechanisms underlying the final dataset. For this purpose, we performed the classical Missing Completely at Random (MCAR) test [44] at different levels of analysis: within subjects, between subjects, within days and between days. The MCAR test evaluate the null hypothesis that data are missing completely at random. In line with [45], we set a *p*-value < 0.05 for rejecting the MCAR basic assumption. If this assumption can be retained for all levels of analysis, substantive models can be reasonably analyzed by handling missing data with the Full Information Maximum Likelihood approach (FIML [46]).

Consistent with our theory, we tested an ESM (multilevel) path analytic model. This model was tested with Mplus 8.5 [47]. At the within (*daily fluctuations*) level, the time-lagged PC (PC_t−1_) was specified as associated with Reward Anticipation_t−1_ and both variables, in turn, predicting the unlagged version of Activity Pleasantness (Activity Pleasentness_t_). Moreover, Activity Pleasantness_t_ was controlled for previous levels of the same variable (Activity Pleasantness_t−1_), but also for minutes to midnight associated with each time point of assessment as an additional time-varying covariate. Moreover, the interaction between PC_t−1_ and Reward Anticipation_t−1_ and PC_t−1_ and Activity Pleasantness_t−1_ in explaining Activity Pleasantness_t_. All independent variables and Activity Pleasantness_t−1_ were previously centered around the participant mean (see [48]), and the scores of the last time point of daily assessment of the lagged variables were not allowed to predict the first score in Activity Pleasantness_t_ of the subsequent day (see [28]). At the between level (*subjects*), intercepts of dependent variables and slopes were treated as random, and the between-level covariance matrix was set to unstructured.

## 3. Results

### 3.1. Descriptives

As shown in Table 1, the two groups did not differ for any of the examined baseline sociodemographic, dispositional and state variables.

### 3.2. Efficacy of the Experimental Manipulation

**Figure 1:**
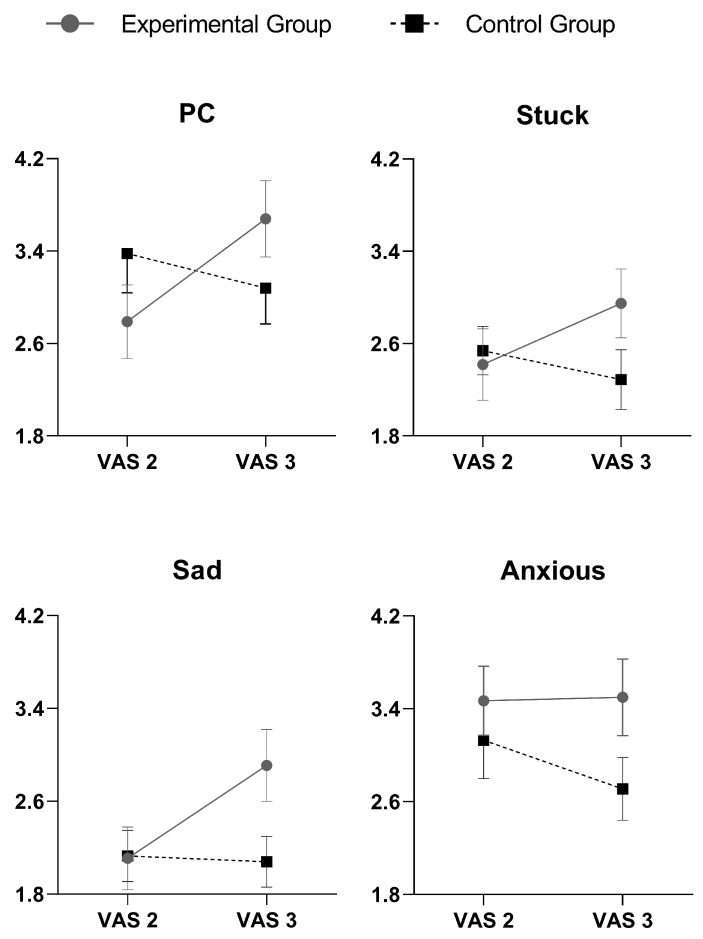
Effects of perseverative cognition induction on momentary mood and levels of state perseverative cognition (PC) as assessed by visual-analog scales (VAS) in the experimental (*n* = 22) and control groups (*n* = 24). VAS 2, pre induction; VAS 3, post induction. Error bars indicate mean standard errors.

The independent sample t-tests revealed significant group differences for state levels of: (1) PC (*t*_(41)_ = 2.79, *p* = 0.008, *d* = 0.87); (2) being stuck (*t*_(41)_ = 3.07, *p* = 0.004, *d* = 0.96); and (3) being sad (*t*_(41)_ = 2.62, *p* = 0.012, *d* = 0.82). There was a stronger increase from pre- to post-manipulation in all these variables in the experimental compared to the control group (see Figure 1). When we evaluated whether these effects were prolonged until the end of the second PRT, no significant group differences emerged, indicating no changes in any of the examined variables from post-manipulation to the end of the protocol.

### 3.3. Performance on the PRT

**Figure 2:**
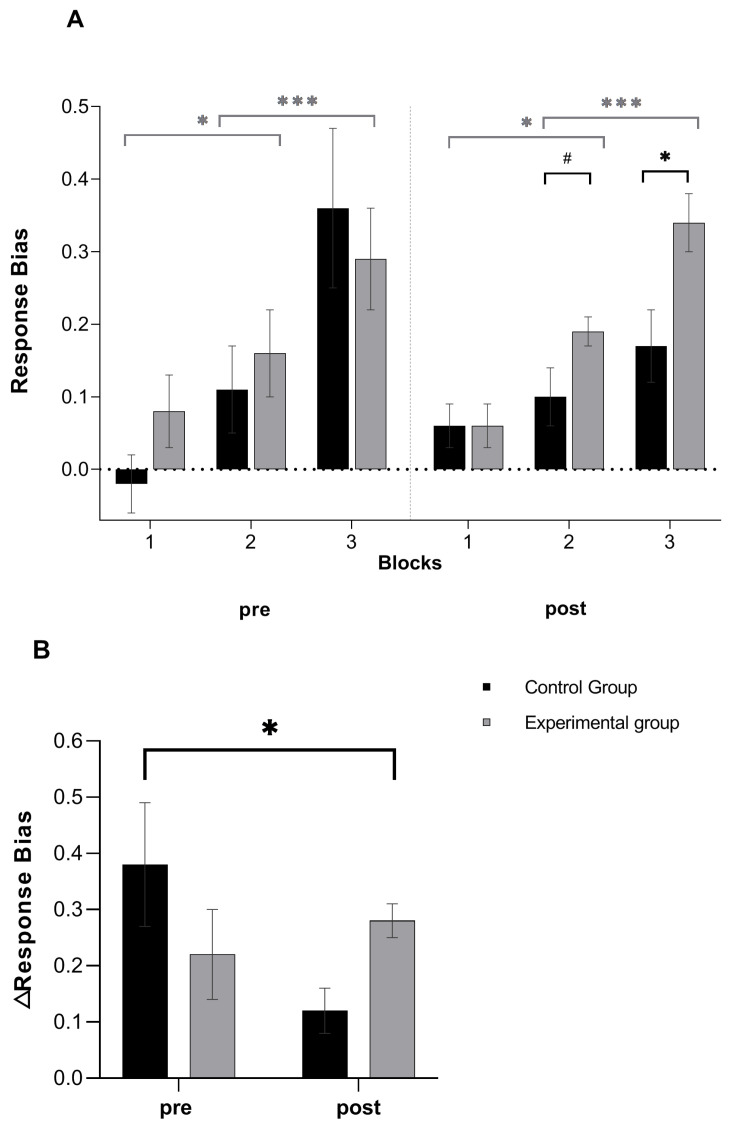
Effects of perseverative cognition induction. Pre-to-post induction changes in Response Bias (**A**) and ΔResponse Bias (**B**) in the experimental (*n* = 22) and control groups (*n* = 24). Error bars indicate mean standard errors. *** *p* < 0.0001, * *p* < 0.05, ^#^ *p* < 0.06.

Huynh–Feldt correction was applied due to violation of sphericity assumption. The GLM with Response Bias as the dependent variable revealed a main effect of Block (*F*_(1.61, 70.7)_ = 33.33, *p* < 0.0001, η_p_^2^ = 0.043) and a significant Time X Group X Block interaction (*F*_(1.77, 77.8)_ = 3.78, *p* = 0.031, η_p_^2^ = 0.079). For the main effect of Block, Bonferroni-corrected post-hoc analysis showed a progressive increase in response bias from the first block to the third block, where Block 1 was characterized by a significantly lower response bias compared to Block 2 (*d* = 0.46, *CI* = −0.16, −0.03, *p* = 0.007) and Block 3 (*d* = 1.19, *CI* = −0.34, −0.15, *p* < 0.0001), with Block 2 being characterized by a lower bias than Block 3 (*d* = 0.73, *CI* = −0.23, −0.08, *p* < 0.0001). For the three-way interaction, Bonferroni-corrected post-hoc analysis of simple effects showed that the experimental group had a significantly higher post-manipulation response bias in Block 3 (*d* = 0.84, *CI* = 0.23, 1.44, *p* = 0.007) and a marginally significantly higher post-manipulation response bias in Block 2 (*d* = 0.52, *CI* = −0.01, 0.1, *p* = 0.080) relative to the control group (see Figure 2A).

Moreover, the GLM having ΔResponse Bias as the dependent variable, revealed Time X Group interaction (*F*_(1, 46)_ = 5.50, *p* = 0.024, η_p_^2^ = 0.11), where Bonferroni-corrected post-hoc simple effects analysis showed higher ΔResponse Bias from pre- to post-manipulation in the experimental relative to the control group (*d* = 0.95, *CI* = −0.02, −0.31, *p* = 0.015) (see Figure 2B).

Analyses on discriminability scores showed a main effect of Time (*F*_(1, 44)_ = 20.64, *p* < 0.0001, η_p_^2^ = 0.31), with reduced discriminability during performance on the second compared to first PRT (*d* = 0.67, *CI* = 0.12, 0.34, *p* < 0.0001), suggesting that the second PRT (nose version of the task) was more difficult for both groups, along with a main effect of Block (*F*_(2, 88)_ = 6.05, *p* = 0.003, η_p_^2^ = 0.12), showing increased discriminability in Block 2 compared to Block 1 (*d* = 0.38, *CI* = 0.01, 0.12, *p* = 0.022) and in Block 3 compared to Block 1 (*d* = 0.49, *CI* = 0.20, 0.14, *p* = 0.004). No significant Group X Time interaction (*F*_(2, 88)_ = 1.65, *p* = 0.21) or Time X Group X Block interaction emerged (*F*_(2, 88)_ = 0.02, *p* = 0.98) (see Figure S2A).

Total accuracy showed main effect of Time (*F*_(1, 44)_ = 79.54, *p* < 0.0001, η_p_^2^ = 0.33), with reduced overall accuracy during performance on the second PRT in both groups (*d* = 1.31, *CI* = 0.12, 0.19, *p* < 0.0001) (see Figure S2B). No main effect of Group or significant interactions (*F*_(1, 46)_ = 0.47, *p* = 0.50) emerged (see Supplementary Materials for further control analyses).

### 3.4. Computational Modeling

**Figure 3:**
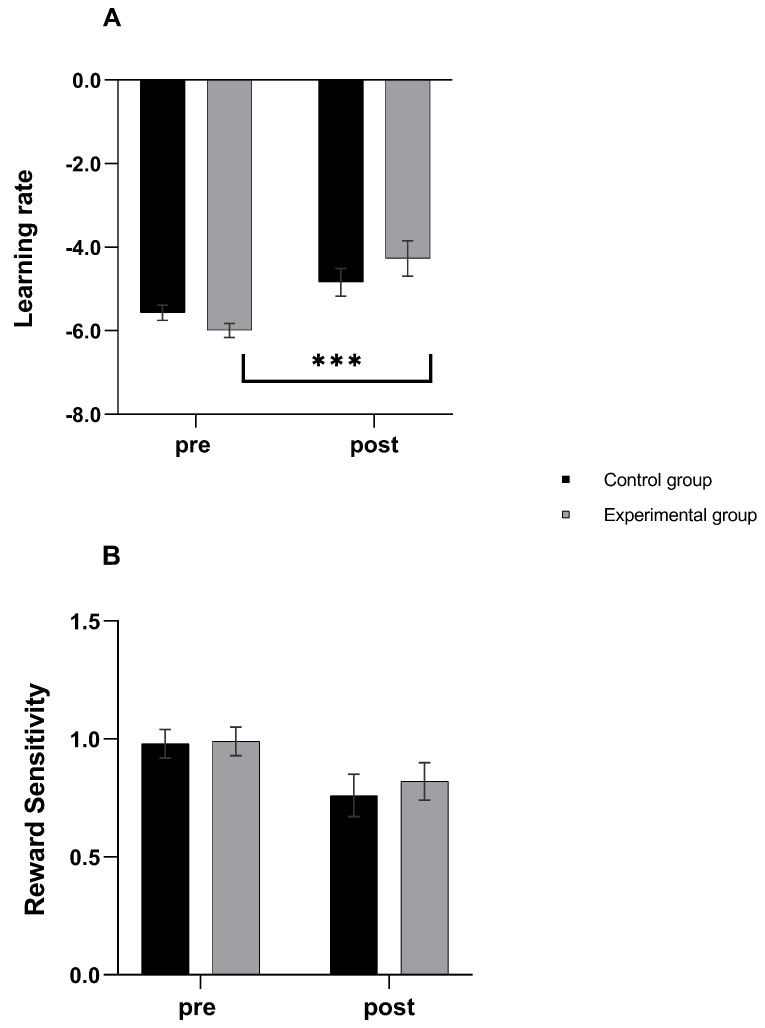
Changes in computational modeling parameters. Pre- to post-induction changes in Learning rate (**A**) and Reward sensitivity (**B**) in the control and experimental groups. Error bars indicates mean standard errors. *** *p* < 0.001. Note that parameters were computed in the transformed space in order to prevent issues with non-Gaussianity. Hence, they are both unconstrained in range and larger values indicate greater Learning rate and Reward sensitivity.

The two-way GLM on learning rate yielded a significant main effect of Time (*F*_(1, 44)_ = 21.76, *p* < 0.0001, η_p_^2^ = 0.33) and a marginally significant Time X Group interaction (*F*_(1, 44)_ = 3.58, *p* = 0.06, η_p_^2^ = 0.075). As depicted in Figure 3, post-hoc comparisons revealed that learning rate increased in the second PRT only in the experimental group (*d* = 1.15, *CI* = 0.54, 1.76, *p* < 0.0001). For reward sensitivity, a main effect of Time emerged (*F*_(1, 44)_ = 7.23, *p* = 0.010, η_p_^2^ = 0.14), with a significant decrease from the first to the second PRT (*d* = −0.40, *CI* = −0.33, −0.48, *p* = 0.010) in both groups.

### 3.5. EMA

**Figure 4:**
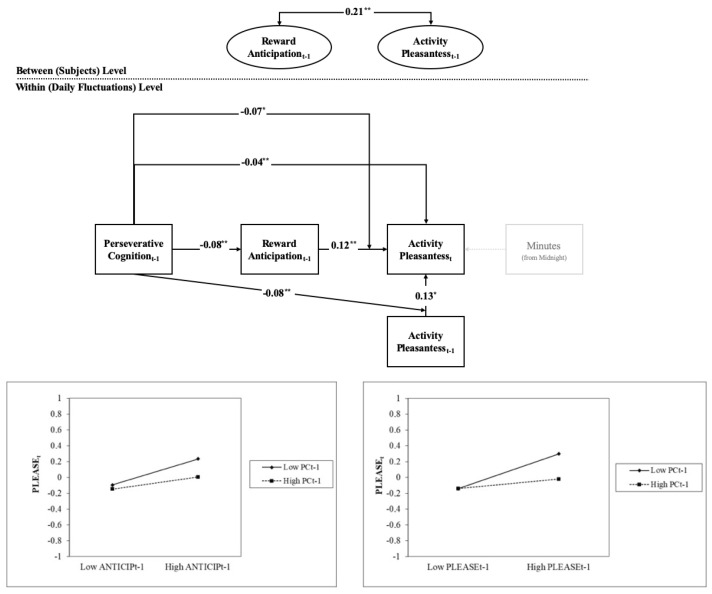
The Empirical ESM Path Analytic Model. (**top**) All parameters represent Beta coefficients expressed in a completely standardized metric. Dotted lines indicate non-significant effects. ** *p* < 0.01, * *p* < 0.05 (**bottom**) Plot of the Significant Interactions from the Empirical ESM Path Analytic Model. The results are presented in a completely standardized metric. PLEASE_t_ = Activity Pleasantness; PC_t−1_ and ANTICIP_t−1_ = time-lagged version of Perseverative Cognition and Reward Anticipation.

MCAR tests were not statistically significant for all levels of analysis, suggesting that missing data were unrelated to the study variables, specific days and time points of assessment for all participants. Thus, the following ESM path analytic model was tested by handling missing data with the FIML approach and by using robust maximum likelihood estimators (MLR; see [47]). Since after a first model run all random slopes parameters were not statistically significant, they were all fixed to zero. Considering the high level of correlation between self-reported levels of Reward anticipation and Effort (*r* = 0.944; *p* < 0.001), a composite score (from now on referred to as Reward Anticipation) was created by summing the scores of their relative items per participants and time point of assessment. Model fit with observed data was close to perfect: MLR$\chi^{2}$ = 1.890_(df=4)_, *p* = 0.756; Root Mean Square Error of Approximation (RMSEA) = 0: Comparative Fit Index (CFI) = 1.00; Tucker–Lewis Index or Non-normed Fit Index (TLI or NNFI) = 1.00; *within-level* Standardized Root Mean Square Residual (SRMR) = 0.022; *between-level* Standardized Root Mean Square Residual (SRMR) = 0. Standardized results of the tested ESM path analytic model are presented in Figure 4.

Specifically, an increase in time-lagged PC_t−1_ was significantly and negatively associated with time-lagged Reward Anticipation_t−1_. Activity Pleasantness_t_ was significantly and positively predicted by the time-lagged version of the same variable and Reward Anticipation_t−1_, while it was significantly but negatively predicted by PC_t−1_. Moreover, the stable (between-subjects) components of the time-lagged version of Reward Anticipation and Activity Pleasantness were significantly and positively correlated. Finally, the interaction between time-lagged PC and Activity Pleasantness and PC and Reward Anticipation in predicting Activity Pleasantness were both significant and negative. Specifically, low levels of PC_t−1_ produced an additional increase in Activity Pleasantness_t_ scores under conditions of, respectively, high Reward Anticipation_t−1_ and Activity Pleasantness_t−1_.

## 4. Discussion

To date, PC has been almost exclusively studied within the negative valence systems. This is somehow surprising if we consider that PC is now recognized as a transdiagnostic factor, which is present across psychiatric disorders, such as substance use disorder or in the manic phase of bipolar I disorder (e.g., [14,49]). To fill this gap, and in line with a recent call to study transdiagnostic factors both across and within different RDoC domains [16], the present study combined an experimental paradigm with experience sampling in daily life with the goal of providing an ecologically valid view of the effects of PC on reward learning. To do so, in the experimental session, we used a task designed to objectively assess participants’ ability to modulate behavior as a function of reward [17] before and after the induction of PC.

### 4.1. Perseverative Cognition, Stress Response and Reward Functionality

Contrary to our hypotheses, results point to a higher response bias toward the most frequently rewarded stimulus in the experimental compared to the control group, suggesting that PC enhanced reward responsiveness and the ability to shape future behavioral choices based on prior reinforcement experiences. Considering that PC is usually associated with a significant physiological stress-response (i.e., fight or flight) (see [20] for a meta-analysis), the current finding is somehow inconsistent with previously reviewed results on the effects of an acute stressor (e.g., threat of shocks) on reward responsiveness, where stress-related blunted reward responsiveness and reward learning have been reported [18,19]. This discrepancy might have occurred due to differences in stress paradigms such as type of induction (e.g., threat-of-shock and PC induction) and in stress-to-task latency (e.g., during the task and just before the task). For example, relative to the type of induction, Ottaviani and colleagues [20] noted that the physiological correlates (e.g., systemic cortisol release) of PC and acute stressor are different in terms of quantity and duration. Although the magnitude of the effect of acute stress is greater in terms of physiological activation, PC activates the body more frequently and for longer time, making the existence of different pathways to alteration of reward processing functionality possible. Relative to stress-to-task latency, different cellular and neuroendocrine dynamics occur on the ground of stressor onset (e.g., [50]). Indeed, administering the reward learning task just before—instead of during—the stress manipulation seems to be associated with an increase in reinforcement learning [51,52]. Notably, in the current study, the PRT was administered immediately after the induction and the subjective effects of PC induction are maintained until the end of the task, as shown by the visual-analog scales.

It is also important to note that our finding of a potentiated effect of PC on reward functionality is not without precedents. Whitmer and colleagues [24] found that a depressive rumination induction just before the probabilistic reward selection task, a reinforcement learning task similar to the PRT, led to a higher sensitivity to reward relative to a distraction induction in both clinically depressed and healthy individuals. Moreover, the dispositional tendency to ruminate was found to be positively associated with activation of the ventral striatum regions and increased connectivity within cortico-striatal circuits in response to rewards [22]. This finding is somewhat contrary to results of a recent meta-analysis of positive valence system functionality in depressive patients, where the largest impairment emerged precisely in the subconstruct of reward learning [53].

In an attempt to understand whether the greater reward bias triggered by PC was due to the effect of learning rate (i.e., the ability to learn from reward feedback) or to reward sensitivity (i.e., the hedonic immediate behavioral impact of rewards), a computational modeling analysis was performed (e.g., [40,54]). The computational modeling highlighted a marginally significant contribution of learning rate (of large effect size) and a non-significant effect of reward sensitivity to the effects of PC on reward processing. Such parameters were derived from the mathematical models of reinforcement learning [55,56]. Specifically, learning rate quantifies the extent to which reward prediction errors (i.e., the difference between the obtained and expected reward) affect learning, specifically the speed to which reward affects behavior [40]. This implies that PC may increase the behavioral impact of prior reward feedback on the current trial-by-trial decision and thus learning in function of prior reinforcements without a concomitant increase of hedonic impact of rewards.

In line with the current finding, evidence suggests that acute stress has dissociable effects on the distinct components of reward processing, particularly potentiating motivation/‘wanting’ during the anticipatory phase of reward processing, while reducing ‘liking’ during the consummatory phase (reward receipt/delivery phase). For example, an acute stress manipulation in the form of performance negative feedback (thought to elicit PC), increased neural and behavioral activation in the anticipatory phase of reward assessed by monetary incentive delay task, designed for the simultaneous measurement of the anticipation and consumption phase of reward processing [57]. Consistently, the dispositional tendency to engage in rumination (i.e., trait rumination) positively correlated with activation of the areas implicated in the salience network to reward—but not to loss—cues in the anticipatory phase of reward processing, after controlling for age, sex, and depressed mood [58].

In the context of more basic investigations, a multitude of animal studies [59,60,61] and some human studies [62] suggest that acute stress manipulation, possible through an increase of brain levels of glucocorticoids and catecholamines [63], quickly activates mesocorticolimbic dopaminergic neurons [59,60,62,64] which in turn potentiates cue-triggering wanting, attentional orienting toward salient events and learning from behavioral reinforcements [61,65].

### 4.2. Perseverative Cognition and Daily-Life Motivational System Functionality

A more ecological assessment of behaviors and cognition outside the laboratory has been called for to draw more valid conclusions on mental health functioning [42]. In line with this, we combined an ecological assessment of daily-life occurrence of the cognitive processes underling PC with the measurement of everyday reward-related behavior, to enhance the validity and understanding of laboratory results.

Interestingly, the momentary occurrence of PC modulated the association between the anticipation of a reward and the concurrent tendency to be actively engaged with and satisfied by such reward. In other words, in moments when participants were stuck in their thoughts, they appeared to be less sensitive to rewards value even when they had been showing high levels of anticipation and motivation to pursue them. Such an observed decoupling between the anticipation and the consummatory phase of reward processing during episodes of PC generates a discrepancy between the expected and obtained reward, that is a prediction error. Considering that learning rate, as assessed by the applied computational modeling, particularly incorporates measures of prediction error [40], it is plausible that the laboratory finding of an increased learning rate during performance on the PRT following PC might derive from an increase in reward prediction error. This interpretation would be consistent with the common idea that maladaptive PC comes from a recurrent and unfruitful attempt to reduce the discrepancies between actual and desired goals [13,66]. If PC exacerbates such discrepancy, as suggested by both current laboratory and ecological results, this could be a mechanism through which this maladaptive process is pathogenically maintained. In support of this speculative hypothesis, Ditcher and colleagues [67] found greater reward network activation during the anticipatory phase of reward processing paired with reward network hypoactivation during reward outcomes in individuals with major depression; in interpreting such results, the authors point to the role of rumination.

Besides potentiating reward-related prediction error, when the anticipatory and the consummatory phases of reward were examined separately, daily occurrence of PC had the effect to dampen each of these components. Notably, the occurrence of PC not only reduced the hedonic value of a concomitant reward but also buffered the positive effect of such reward on the subsequent one. This is well in line with the fact that depressed patients who are characterized by higher levels of PC are more prone to relapse after psychological or pharmacological treatment [68].

## 5. Limitations and Conclusions

The current results should be interpreted with due consideration of the limitations of the study. First, data were collected by setting up a remote experimental session due to SARS-CoV-2 pandemic. Noteworthy, the required methodological rigor was maintained by having the experimenter connected online throughout the experimental protocol. However, extra-experimental environmental variables, such as performing the task with one’s personal computer, may have biased the results. In regard to this, we decided not to use reaction times as a control variable of the behavioral output of the PRT. The analysis of the reaction times could have informed us on the speed of choice of the stimulus that guided the learning, therefore providing further indication on the development of bias in the two groups. Despite this, the specificity of the measurement of the response bias was supported by control analysis such as the analysis of discrimination and accuracy. Second, it is possible that the historical period during which the study was performed could have influenced some of the EMA variables, for example the assessment of reward-related behavior during times of limited freedom. The COVID-19 pandemic has led to widespread increases in mental health problems, including anxiety and depression, and this might have intensified the daily occurrence and severity of PC episodes. Even though we have no reasons to expect that the global health situation has specifically affected the association between PC and reward-related behaviors, it would be important to replicate the EMA results on different samples after the end of the pandemic. Third, the sample size is limited, mostly composed of university students, and unbalanced with regards to gender distribution. Given the well-known gender differences in the tendency to develop hypo-motivational (e.g., depression) and hyper-motivational (e.g., addiction) disorders, future studies should investigate the moderating role of gender on the association between daily PC and reward-based learning.

To conclude, the experimental induction of PC in the laboratory potentiated reward-based learning, and ecological data suggest that this may derive from potentiated reward prediction error, due to a PC-induced decoupling between the anticipatory and consummatory phase of reward. Together with the negative consequences on the consummatory phase of reward, likely leading to a long-lasting reduced hedonic impact of rewards value, the hereby reported effects on prediction error may inform maintenance mechanisms. For example, it has been shown that PC may render individuals more prone to engage in unhealthy habitual behaviors, such as food or alcohol binge intake [69,70].

Present data advise not only toward treating PC as a dimensional and transdiagnostic construct [71] but also toward the implementation of ad hoc interventions to normalize positive valence systems (dis)functionality. To maximize the clinical application of current findings and gather a more mechanistic understanding, it would be important to investigate neurobiological alterations underpinning reward processing dysfunctions during PC. Alterations within mesocorticolimbic circuits, commonly thought to be implicated in reward processes and in motivational deficits in psychiatric disorders, have not been specifically associated with PC. However, emerging evidence exists, pointing to alterations in corticostriatal functioning associated with both state and trait PC [22,58,72]. In line with the proposal of Kalivas and Kalivas [73], further investigations of the corticostriatal involvement in the maintenance of PC and its effects on reward-related behavior could forecast therapeutic impact on many disorders, all afflicted by this maladaptive symptom.

## Figures and Tables

**Table 1:** Mean and standard deviations for state, trait and sociodemographic variables at baseline. PTQ, Perseverative Thinking Questionnaire; SHAPS, Snaith–Hamilton Pleasure Scale; STAI, StateTrait Anxiety Inventory; CESD, Center for Epidemiological Studies Depression Scale; OS, Optimism Scale; BIS_NP, Non Planning subscale of the Barratt Impulsiveness Scale; BIS_Motor, Motor subscale of the BIS; BIS_Cognitive, Cognitive subscale of the BIS; BAS_drive, Drive subscale of the Behavioral Activation System; BAS_FunSeek, Fun Seeking subscale of the Behavioral Activation System; BAS_RewResp, Reward Responsivity subscale of the Behavioral Activation System; BIS, Behavioral Inhibition System. VAS 1, Visual-Analog Scale administered at baseline, PC, Perseverative Cognition. * value output of Mann–Whitney test for non-parametric distributions.

Variables	Controls *n* = 24	Experimental Group *n* = 22	*t*/*U* *	*p*
Age	21.9 (2)	24 (7.5)	244 *	0.66
PTQ	29.4 (11.1)	28.3 (11)	0.24	0.74
SHAPS	15 (7.7)	13 (1.5)	1.36	0.23
STAI-T	42.5 (13.4)	43.4 (12.2)	0.26	0.86
CESD	20.9 (8.2)	20.3 (6.2)	0.28	0.77
OS	31 (4.9)	30.9 (6)	0.13	0.95
BIS_NP	2.2 (0.4)	2.1 (0.3)	0.81	0.40
BIS_Motor	1.7 (0.3)	1.7 (0.3)	0.44	0.53
BIS_Cognitive	1.9 (0.4)	2.0 (0.4)	0.80	0.52
BAS_Drive	12.5 (1.3)	12.6 (1.3)	0.44	0.81
BAS_FunSeek	12 (1.6)	12.5 (1.3)	1.20	0.30
BAS_RewResp	15 (1.7)	15 (2)	0.77	1
BIS	20.1 (2.1)	20 (2.1)	0.39	0.89
Boredom	3.4 (1.3)	3.3 (1.8)	0.14	0.83
Disinhibition	4.2 (2.3)	4.5 (2.1)	0.41	0.76
Thrill Adv. Seeking	6.6 (2.5)	6.9 (2.8)	0.34	0.72
Experience Seeking	6.5 (1.9)	6.7 (1.3)	0.47	0.71
**VAS 1**				
PC	3.2 (1.5)	3.5 (1.3)	0.66	0.49
Stuck	2.6 (1.2)	2.9 (1.2)	0.85	0.37
Happy	4.4 (1)	4.9 (1)	1.79	0.08
Anxious	3.4 (1.6)	3.7 (1.6)	0.57	0.51
Satisfied	4.7 (0.9)	4.5 (1.1)	0.49	0.41
Sad	2.3 (1.1)	2.3 (1.2)	0.36	0.85
Enthusiastic	3.7 (1.4)	4.3 (1.3)	1.90	0.13
Energetic	3.9 (1.1)	4.4 (1.4)	1.68	0.19
			$\chi^{2}$	
Sex	19F, 5M	16F, 6M	0.26	0.60

## Data Availability

The data presented in this study are available upon request to the corresponding author.

## Supplementary Materials

The following are available online at https://www.mdpi.com/article/10.3390/brainsci11050585/s1, Supplementary Methods: Procedure, Perseverative cognition induction, Probabilistic Reward Task (PRT), Computation Modeling, Quality Control (QC) cutoffs for PRT data; Supplementary Results: Accuracy analyses; Supplementary references; Supplementary figures: Figure S1: Experimental timeline, Figure S2: Control analysis, S2A: Discriminability scores toward the blocks in control and experimental group pre to post induction, S2B: Total accuracy in control and experimental group pre to post induction, S2C: Rich Accuracy and S2D: Lean Accuracy in control and experimental group pre to post induction.

## Abbreviations

The following abbreviations are used in this manuscript:

RDoC	Research Domain of Criteria
PC	Perseverative Cognition
PRT	Probabilistic Reward Task
EMA	Ecological Momentary Assessment
ESM	Experience Sampling Method
VAS	Visual-Analog Scale
GLM	General Linear Model
MCAR	Missing Completely at Random
FIML	Full Information Maximum Likelihood
