# Supplementary material for: Perseverative Cognition in the Positive Valence Systems: An Experimental and Ecological Investigation
Source: Brain Sci. 2021 Apr 30;11(5):585. doi: 10.3390/brainsci11050585 (PMC8147166; doi:10.3390/brainsci11050585)
Supplement: Supplementary file 1 [file brainsci-11-00585-s001.zip › brainsci-1204532-supplementary.pdf]

Perseverative cognition in the positive valence systems: An experimental and ecological investigation

Martino Schettino, Valerio Ghezzi, Yuen-Siang Ang, Jessica M. Duda, Sabrina Fagioli,

Douglas S. Mennin, Diego A. Pizzagalli, Cristina Ottaviani

Supplementary Methods

Procedure

Participants received a link to an online appointment with the experimenter via Hangouts Meet. Before beginning the session, participants were instructed via email to download the Inquisit player installer to run the whole script of the experiment. Participants were required to perform the experiment alone, in a silent room and to turn off any electronic device nearby (e.g., mobile phone or sounds of own personal computer). The experimental session was conducted entirely on Hangouts meet. Before starting, the experimenter made sure that the environment in which the experiment took place was adequate. To maintain the required methodological rigor during the experimental protocol, participants were observed while they performed the task, and they verbally interacted with the experimenters during the instructions and experimental manipulation phases. See Fig. S1 for the experimental timeline.

Perseverative cognition induction

Most participants recalled an episode about their past, with prototypical examples being termination of a significant relationship or exam failure.

Probabilistic Reward Task (PRT)

For the present study, the script of the Millisecond version of the PRT was adapted following the procedures detailed in the manual from Pizzagalli and colleagues (Pizzagalli & Whitton, 2020). With

the exception of the version of the PRT used (mouth vs. nose), which could not be counterbalanced due to limitations associated with the Inquisit Web licence (https://www.millisecond.com/products/inquisit6/weboverview.aspx), within- and between-subjects counterbalancing occurred on the basis of 1) the keyboard key that was mapped to the rich and lean stimuli, and 2) the amount of money the subject received in total.

The adapted version of the task consisted of 100 trials per 3 blocks with a total of 300 trials. Blocks were separated by a 30-second break. Trials began with a fixation cross for 1400 ms in the middle of the screen. The cross was replaced for 500 ms by a mouthless or noseless face, respectively in the mouth or nose version of the task. After 500 ms, in the mouth version of the task, a short mouth (10.00 mm) or a long mouth (11.00 mm) was presented; in the nose version of the task, a short (5.00 mm) or a long nose (5.31 mm) was presented. After 100 ms, the mouthless or noseless face returned and remained on the screen for additional 1500 ms. Participants were instructed to identify which stimulus (long or short) was presented by pressing one of two button responses ("E" or "I") on the keyboard. The button responses were counterbalanced within subjects (for the mouth and nose versions) and across subjects. Participants were informed that the goal was to win as much money as possible with the indication that not all correct responses would be rewarded. Within each block, an equal number of short and long stimuli was presented in a pseudo-randomized sequence with the constraint that no more than three instances of the same stimulus would be presented consecutively. For each block an asymmetric 3:1 reinforcement ratio was applied: i.e., in the case of a correct response, a reward feedback ("Correct!! You won 20 cents") was presented 3 times more for one stimulus (the so-called rich stimulus) than for the other (the so-called lean stimulus). During each block, each participant received 30 reward feedbacks for correct identification of the rich stimulus and only 10 reward feedbacks for correct identifications of the lean stimulus. The reward feedback was given according to a controlled reinforcer procedure that determined in a pseudorandom way which specific trials were to be rewarded for correct identifications. If a

participant failed to make a correct response for a trial in which feedback was scheduled, reward feedback was delayed until the next correct identification of the same stimulus type (rich or lean). The reward feedback (1500ms) was replaced by a blank screen for 250 ms before the new trial. If feedback was not given a blank screen was displayed for 1750 ms.

Computational Modelling

We conducted hierarchical/random effects estimation via Expectation Maximization with Laplace's Approximation for the whole sample under the same prior. This is because the individual subject parameters that are estimated would no longer be independent and thus, the individual subject parameters need to be derived under the same prior in order for us to apply standard statistical tests, such as correlations and ANOVAs, for further analyses. This procedure was well-established by Huys et al. (2013) who estimated patients with Major Depressive Disorder, euthymic patients, and healthy volunteers who took a single dose of placebo or pramiprexole, as well as healthy people who underwent both an acute threat-or-shock stressor and no stress, together under the same prior; and has been applied in subsequent publications such as Pizzagalli et al. (2020), who used depressed patients on placebo vs. kappa opioid receptor antagonist; and Whitton et al. (2020), who used depressed patients and healthy controls.

Because different versions of the task were used for pre-manipulation (mouth version) and post-manipulation (nose version), we also conducted additional analyses at the request of an anonymous reviewer by re-running the computational modelling separately for pre- and post-intervention and verified that the 'Action' was still the best-fitting for both pre-manipulation (group-level log Bayes factor of 48 over the second-best 'Belief' model, which represents very strong evidence) and post-manipulation (group-level log Bayes factor of 18 over the second-best 'Belief' model, which represents strong evidence). Importantly, the parameters derived from these additional analyses were highly correlated with the original parameters obtained by treating the whole sample

together (rreward sensitivity=0.951, rlearning rate=0.972, rinstruction sensitivity=0.989, rinitial bias=0.930), suggesting that the likelihood was strong and there was little difference in the parameter values obtained by considering the whole sample together vs. treating pre- and post- separately.

Quality Control (QC) cutoffs for PRT data

Quality control check was performed, blind to group assignment, following the indication of procedures manual. To identify usable PRT data, QC cutoffs were:

- ≥ 80% number of valid trials for each block, i.e., < 20 outliers per block that included (a) reaction time (RT) on each trial shorter than 150 ms and longer than 2500 ms; and (b) log-transformed RT exceeding the participant's mean of ± 3 SD;
- rich-to-lean reward ratio in each block of ≥ 2.0;
- the number of rich and lean rewards in each block ≥ 20 and ≥ 6, respectively.

Given that the nose version of the task was more difficult than its mouth version and validation procedures were established for the mouth version, less stringent exclusion criteria were applied for the nose version. For the mouth version of the task, participants were excluded from the analyses even if one out of three QC criteria was not fulfilled. Differently for the nose version of the task, participants were excluded from the analyses when two out of three QC criteria were not fulfilled.

Supplementary Results

Accuracy analyses

The analysis with total accuracy as dependent variable was followed-up by examining the effects of induction on lean and rich accuracy, separately. Relative to rich accuracy, the GLM yielded a main effect of time ($F(1, 44) = 61.94$, $p < .0001$, $\eta_p^2 = .52$) but no main effects of group ($F(1, 44) = 1.65$, $p = .21$) or significant interactions emerged ($F(1, 44) = .005$, $p = .94$). Bonferroni post-hoc analysis revealed a reduced accuracy for the rich stimuli post-manipulation for both groups

(d =1.16, CI = 0.11, 0.19, $p < .0001$). Relative to lean accuracy, the GLM yielded a main effect of time ($F(1, 44) = 49.21$, $p < .0001$, $\eta_p^2 = .23/.52$) but no main effects of group ($F(1, 44) = 0.01$, $p = .93$). or significant interactions ($F(1, 44) = 1.08$, $p = .30$). Bonferroni-corrected post-hoc analysis revealed a reduced accuracy for the lean stimuli for both group (d = 1.03, CI = 0.11, 0.20, $p < .0001$) (see Fig. S2 panel C and D).

Supplementary references

Pizzagalli, D. A., Smoski, M., Ang, Y.-S., Whitton, A. E., Sanacora, G., Mathew, S. J., Nurnberger, J., Lisanby, S. H., Iosifescu, D. V., Murrough, J. W., Yang, H., Weiner, R. D., Calabrese, J. R., Goodman, W., Potter, W. Z., & Krystal, A. D. (2020). Selective kappa-opioid antagonism ameliorates anhedonic behavior: Evidence from the Fast-fail Trial in Mood and Anxiety Spectrum Disorders (FAST-MAS). Neuropsychopharmacology, 45(10), 1656–1663. https://doi.org/10.1038/s41386-020-0738-4

Pizzagalli, D. A., & Witton, A. (2020). Probabilistic Reward Task or “Face Game. Procedures manual for obtaining behavioral and event-related potential data. Updated: February 10, 2020.

Whitton, A. E., Reinen, J. M., Slifstein, M., Ang, Y.-S., McGrath, P. J., Iosifescu, D. V., Abi-Dargham, A., Pizzagalli, D. A., & Schneier, F. R. (2020). Baseline reward processing and ventrostriatal dopamine function are associated with pramipexole response in depression. Brain, 143(2), 701–710. https://doi.org/10.1093/brain/awaa002

Figure S1. Experimental timeline.

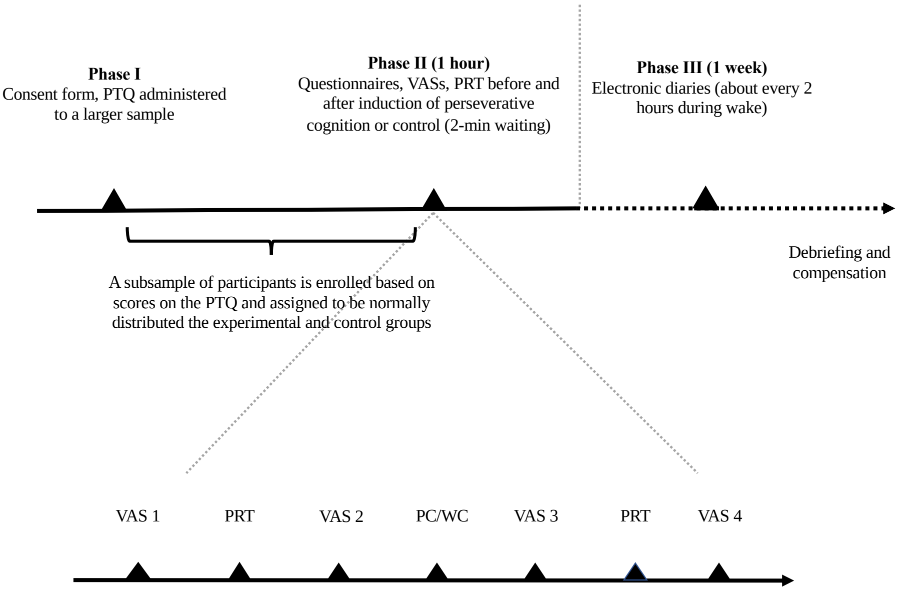

Note. PTQ = Perseverative Thinking Questionnaires; VAS = Visual Analog scales; PRT = Probabilistic Reward Task; PC = Perseverative cognition induction (experimental manipulation); WC = Waiting (control) Condition.

Figure S2. Control analysis. (A) Discriminability scores toward the blocks in control (*n = 24*) and experimental group (*n = 22*) pre to post induction. (B) Total accuracy in control (*n = 24*) and experimental group (*n = 22*) pre to post induction (C) Rich Accuracy and (D) Lean Accuracy in control and experimental group pre to post induction.

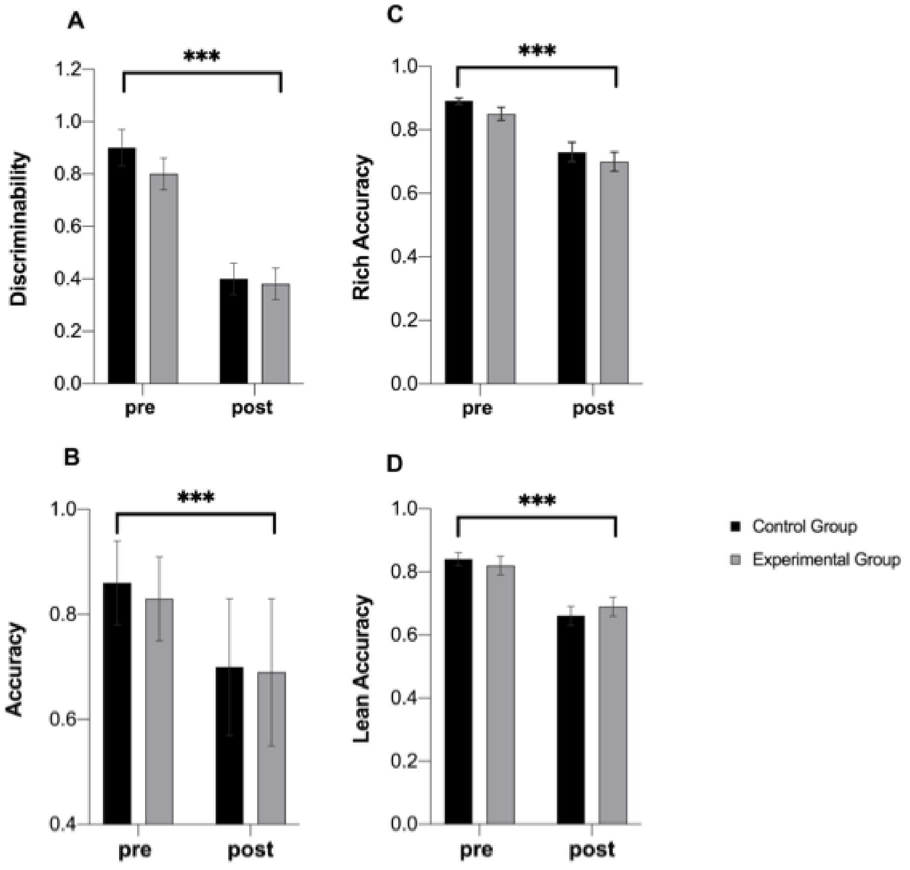

Note. Error bars denote mean standard errors. Estimated means are plotted. Flags denote the main effects of the block variable. ***$p < 0.0001$, **$p < 0.05$
